# Impulsivity and Cognitive Functioning in Inpatients with Concurrent Disorders: A Comparative Study with Healthy Controls and Evaluation of Treatment-Related Changes: Impulsivité et fonctionnement cognitif chez les patients hospitalisés présentant des troubles concomitants : étude comparative avec des témoins sains et évaluation des changements liés au traitement

**DOI:** 10.1177/07067437241303407

**Published:** 2024-12-05

**Authors:** Stefanie Todesco, Thomas Chao, Liam Gorsuch, Christian Schütz

**Affiliations:** 1 Department of Psychiatry, 8166 Institute of Mental Health, University of British Columbia, Vancouver, Canada; 2 BC Mental Health and Substance Use Services Research Institute, Provincial Health Services Authority (PHSA), Vancouver, BC, Canada

**Keywords:** impulsivity, cognitive function, concurrent disorders, inpatient treatment, substance use

## Abstract

Impairments in impulse control and related neurocognitive functioning have been well documented in both substance use and mental disorder populations. While high impulsivity and other executive dysfunction have been linked to negative treatment outcomes in these populations, no study to date has examined their clinical relevance among individuals with co-occurring substance use and mental disorders (or concurrent disorders [CD]).

**Objective:**

This study investigated impulsivity and working memory among CD inpatients across treatment and compared to controls.

**Methods:**

Patients (*N *= 56, *M*_age_ = 38.2, *SD *= 11.7, 17F) and healthy controls (*N *= 50, *M*_age_ = 31.9, *SD *= 10.0, 25F) completed a battery of self-report questionnaires and behavioural tasks assessing working memory and impulsivity (response inhibition, delay discounting, reflection, decision-making). Patients were assessed within 2 weeks of admission (baseline) and at 6 months (follow-up). Controls completed a single session at baseline. Patient demographics, diagnostic status, and treatment outcome (discharge with or without medical advice) were retrieved from medical records.

**Results:**

Group differences in demographics were probed for inclusion as covariates. At baseline, patients had greater self-reported impulsivity on the UPPS-P (negative and positive urgency) and BIS (motor and non-planning), and greater delay discounting than controls. Among patients, there was no association between treatment adherence and working memory, self-report, or behavioural impulsivity, and no change in behavioural impulsivity was observed from baseline to follow-up.

**Conclusions:**

This is the first study to assess impulsivity and working memory in the context of CD treatment. Patients exhibited greater impulsivity on choice-based and various self-report measures. The absence of treatment-related changes in impulsivity and working memory outcomes suggests that conventional treatments may be neglecting to target potentially key areas of functioning. Further research is needed to examine how treatment affects impulsivity and related functions in individuals with CD, and their impact on clinical outcomes.

## Résumé

Les déficiences relatives au contrôle des impulsions et au fonctionnement neurocognitif connexe sont bien documentées dans les populations souffrant de trouble lié à l'usage de substances psychoactives et de troubles mentaux. Bien qu'une impulsivité élevée et d'autres dysfonctionnements exécutifs soient associés à des résultats thérapeutiques négatifs dans ces populations, aucune étude à ce jour n'a examiné leur pertinence clinique chez les personnes souffrant de trouble lié à l'usage de substances psychoactives et de troubles mentaux concomitants (ou troubles concomitants [TC]).

**Objectif:**

cette étude s'intéressait à l'impulsivité et à la mémoire de travail chez les patients hospitalisés atteints de TC, en fonction du traitement et par rapport aux témoins.

**Méthodologie:**

les patients (*N* = 56, âge moyen = 38,2, *écart-type* = 11,7, 17 F) et les témoins sains (*N* = 50, âge moyen = 31,9, *écart-type* = 10,0, 25 F) ont rempli une batterie de questionnaires d'auto-évaluation et de tâches comportementales évaluant la mémoire de travail et l'impulsivité (inhibition de la réponse, dévalorisation des gains futurs, réflexion, prise de décision). Les patients ont été évalués dans les deux semaines suivant leur admission (base de référence) et six mois plus tard (suivi). Les témoins ont été évalués une seule fois, à l'étape initiale de l'étude. Les données démographiques des patients, le diagnostic et le résultat thérapeutique (congé avec ou sans consultation médicale) ont été extraits des dossiers médicaux.

**Résultats:**

les différences démographiques entre les groupes ont été examinées pour être incluses comme covariables. À l'étape initiale, les patients se sont déclarés plus impulsifs sur l'échelle UPPS-P (urgence négative et positive) et sur l'Échelle d'impulsivité de Barratt (impulsivité motrice et impulsivité non planifiée), et présentaient une plus grande dévalorisation des gains futurs que les témoins. Parmi les patients, il n'y avait pas d'association entre l'adhésion thérapeutique et la mémoire de travail, l'auto-évaluation ou les comportements impulsifs, et aucun changement en ce qui concerne les comportements impulsifs n'a été observé entre l'étape initiale et le suivi.

**Conclusions:**

il s'agit de la première étude à porter sur l'évaluation de l'impulsivité et de la mémoire de travail dans le cadre du traitement des TC. Les patients ont fait preuve d'une plus grande impulsivité dans les mesures basées sur le choix et dans diverses mesures d'auto-évaluation. L'absence de changements liés au traitement en ce qui concerne l'impulsivité et la mémoire de travail donne à penser que les traitements conventionnels négligent peut-être de cibler des domaines de fonctionnement potentiellement essentiels. Des recherches supplémentaires sont nécessaires pour examiner comment le traitement agit sur l'impulsivité et les fonctions connexes chez les personnes atteintes de TC, ainsi que leur incidence sur les résultats cliniques.

## Introduction

Comorbidity between mental and substance use disorders (SUDs) is associated with increased disease burden.^1^ The co-occurrence of SUD and mental disorders (or concurrent disorders; CD) is associated with greater symptom severity,^2^ poorer treatment adherence and outcome,^3^ and a higher number of emergency room visits,^4^ rehospitalizations and premature deaths^5^ than either disorder alone. CDs are highly prevalent, with 60% of SUD patients estimated to have at least one co-occurring mental disorder.^6^ While conventional treatments have been increasingly adopting an integrative approach to addressing both SUD and mental illness simultaneously, there has been continued reliance on treatment guidelines informed by single-disorder research.^5^ Research investigating individuals with CD as a single clinical group is needed to better understand the common needs of this clinical population and to inform directions for further advancements in treatment.

Impairments in impulse control have been well documented among individuals with SUDs^7,8^ and other commonly co-occurring psychiatric disorders (e.g., depression, anxiety, and bipolar disorder).^9^ Impulsivity has been implicated in the development and clinical severity of SUDs^7,10,11^ and other psychiatric disorders,^9^ contributing to poor treatment outcomes and adverse health effects. It has been identified as the single strongest predictor of substance use and a core risk factor for SUD among individuals with mental illness.^12^ Among patients, those with CD exhibit greater impulsivity^13–15^ and more cognitive impairments^16^ than those with a single disorder. However, research characterizing impulsivity in this population, especially in comparison to healthy controls, remains limited.^8,17^ Considering the growing evidence that impulsivity may be transdiagnostic across SUDs and various mental illnesses,^8,17^ investigating CD collectively as a clinical group has the potential to generate more ecologically relevant and translatable findings.

Impulsivity as a multidimensional construct comprises several facets that can share little overlap between^18^ and within^18–20^ one another. Self-reported impulsivity, commonly assessed using the Barratt Impulsiveness Scale (BIS)^21^ and UPPS-P^22^ Impulsive Behavior Scale, is weakly correlated to behavioural measures.^18^ Behavioural impulsivity more commonly comprises two domains: *Impulsive action,* assessed via motor responses on inhibition (typically Stop Signal [SST]^23^ or Go/NoGo^24^) tasks; and *impulsive choice*, assessed via decision-making on delay discounting or reward-based (e.g., Cambridge Gambling Task [CGT]^25^) tasks.^8,11,18^ Impulsive action is a core component of cognitive control that operates via top-down processes of executive frontal brain regions, whereas impulsive choice is an interplay of top-down executive and bottom-up striatal processes.^26^ Some evidence suggests these domains differ in their association with substance use outcomes, where impulsive choice may be a stronger predictor of long-term abstinence than impulsive action.^19,27^ A third less commonly recognized component of behavioural impulsivity is *reflection impulsivity* (assessed by the Information Sampling Task [IST]^28^), a tendency to forgo the gathering or consideration of relevant information before decision-making. Similar to other impulsivity measures, reflection impulsivity has been reported to be high among substance users.^28^

Working memory and emotion regulation are aspects of executive functioning that have been closely tied to impulsivity, where both are involved in the origin and control of impulsive behaviours.^29–31^ Impulsivity has been linked to working memory ability, such that low working memory ability is associated with more impulsive delay discounting^32^ and higher self-reported impulsivity.^31^ Impulsivity also shows a mediational role in the relationship between working memory and substance use dependence.^30,31^ Impairments in emotion regulation are commonly observed in SUDs, encompassing dimensions of affect intensity/reactivity, affective modulation, cognitive modulation, and behavioural control.^33^ These deficits are associated with poorer impulse control and have been shown to mediate the relationship between impulsivity and substance use factors.^34,35^ While evidence suggests that these executive functions likely interact with impulsivity-related mechanisms to contribute to substance use behaviours, limited research has assessed their relevance in the context of treatment for CD.

This study examined impulsivity and working memory relative to controls, changes in impulsivity and working memory in CD inpatients between baseline (within the first 2 weeks of admission) and 6 months of integrated tertiary care, and whether impulsivity and working memory would predict treatment outcome (discharge with or without medical advice). We hypothesized that patients would differ from controls on all baseline measures. In patients, we expected that impulsivity and working memory would change over treatment and that baseline measures would predict treatment outcomes.

## Methods

### Participants

This study comprised 56 patients recruited at the Burnaby Centre for Mental Health and Addiction (BCMHA) and 50 healthy controls. BCMHA (now known as the Red Fish Healing Centre) is a 100-bed inpatient treatment facility offering comprehensive psychiatric, psychosocial, and medical care for individuals with CD.^36^ Standard treatment consisted of individual and group psychotherapy, emphasizing harm reduction (leading to abstinence), stepped care, withdrawal management, and relapse prevention for a maximum 9-month stay. All patients underwent comprehensive psychiatric evaluation conducted by licensed medical or mental health professionals at treatment admission. Patients were eligible if they were stable on medications and no longer experiencing any withdrawal symptoms, as confirmed by clinical staff. Healthy controls were university students and volunteers recruited via community advertisements. Mini-International Neuropsychiatric Interviews (MINIs) and self-report questionnaires assessing mental health status, medical history, and drug use were administered to controls by trained research staff. Controls were eligible to participate if they were without a current or chronic mental disorder and a current or past substance use disorder. All participants were between ages 18 and 65 and without a history of traumatic brain injury or neurologic disorder, and uncorrected visual or auditory deficits.

Informed consent was obtained in accordance with procedures approved by the Behavioral Research Ethics Board at the University of British Columbia.

### Self-Reports

The UPPS-P Impulsive Behavior and the Barratt Impulsiveness Scales (BIS) were administered to assess self-reported impulsivity. The UPPS-P acronym represents five factors of impulsive behaviours: “negative urgency,” (lack of) “perseverance,” (lack of) “premeditation,” “sensation seeking,” and “positive urgency.” The BIS is a 30-item scale assessing three sub-scales: the ability to concentrate (“attention”), acting without thinking (“motor”), and being present in the moment without future thinking (“non-planning”). While the overlap between the BIS and UPPS-P is well established,^18^ there is evidence to suggest their effects may be unique in substance users.^37^ The 6-item “impulse” subscale of the Difficulties in Emotion Regulation Scale (DERS)^38^ was administered to assess impulse control during negative mood states. This subscale has demonstrated good internal consistency and has shown to be associated with SUD and other clinical outcomes.^38–40^

### Behavioural Tasks

The Delay Discounting Task (DDT)^41^ is a binary choice paradigm measuring impulsive decision-making. Participants chose between two hypothetical scenarios: a larger delayed reinforcer (e.g., $100 in 1 month) or a smaller immediate reinforcer (e.g., $50 right away). The value of the adjusting option increased successively by 50% when chosen but decreased when forgone. The DDT delays ranged from 1 day to 25 years, with the reference value set at $100, and completed again at $1000. The discounting rate, “Mazur's k,”^42^ was computed and averaged across $100 and $1000 settings. This has been done previously in substance use populations,^43^ and we confirmed a positive correlation between settings in our sample (Spearman's rho = .64, *P *< .001).

Five computerized subtests from the Cambridge Neuropsychological Test Automated Battery (CANTABeclipse^TM^; Cambridge Cognition, Cambridge, UK)^44^ were used to assess behavioural impulsivity and working memory. The Information Sampling Task (IST) was used to assess reflection impulsivity. Participants opened a self-determined number of boxes in a 5 × 5 array to reveal their hidden colour before guessing the majority colour. IST outcome variables were mean probability of correct decision under two separate conditions, where opening a box incurred (“decreasing-win”) or did not incur (“fixed-win”) a point penalty. Bennett et al.'s^45^ Bayesian approach was used to compute IST outcomes, due to overestimation in the original formula. The Affective Go/No-Go (AGN) assesses affective information biases and action restraint. Participants were instructed to press a button in response to words matching the target valence (i.e., positive or negative words) and ignore all other words. Each block consisted of nine individually presented target words and nine distractors. The mean response time on trials answered correctly was recorded for both “positive words” and “negative words.” The Spatial Working Memory Task (SWM) measures individuals’ ability to sustain and monitor spatial information in working memory. Participants searched for blue tokens hidden behind boxes, with instructions to refrain from revisiting boxes they previously emptied. The “between errors” score indicates the number of times the participant returned to a box that they already emptied. This outcome has been shown to be correlated (*r = *−.51) with composite battery scores on tests of executive functions.^46^ The Cambridge Gambling Task (CGT) measures risk-taking behaviour and decision-making. On each trial, participants chose a percentage of their points to wager on whether a yellow token was hidden behind a red or blue box. The ratio of red to blue boxes varied across trials, and bet options appeared in fixed intervals in ascending or descending order. The primary outcome, “risk-taking,” was the average points wagered on trials where the higher probability colour was chosen. The Stop-Signal Task (SST) measures the action cancellation component to the broader response inhibition construct.^47^ The SST and Go/No-Go were both included as they measure distinct components of response inhibition: action cancellation and restraint, respectively.^48^ Stop-signal reaction time (SSRT), or the average time required to successfully inhibit 50% of responses across stop trials, was estimated using a staircase function. SSRT from the last half of the test trials was used as the dependent variable.^47^

## Procedures

Participants were randomly assigned to one of four behavioural task batteries that counterbalanced the IST (decreasing-/fixed-win) and CGT intra-task conditions (ascending/descending). The order of task completion was standardized (DDT, IST, AGN, SWM, CGT, SST) to reduce the likelihood of habituation and mental fatigue. Patients completed these tasks within the first 2 weeks of inpatient admission (baseline) and after 6 months of uninterrupted inpatient treatment (follow-up). Controls underwent a single baseline outpatient session. All self-reports were completed once at the start of the experiment. Patients received a $10 Starbucks gift card at the end of each testing session.

## Statistical Analyses

Due to incomplete or missing data, the following behavioural data were missing among the 56 patients: 4 for DDT and IST, 7 for AGN, 5 for SWM and SST, and 6 for CGT. Among 50 controls, behavioral missing data included: 1 for DDT, 4 for IST, and 2 for SST. Of the 27 patients who completed the 6-month follow-up, one was missing DDT and SWM, two were missing SST, and three were missing CGT and AGN data.

Chi-square or independent samples *t*-tests were used to assess demographic differences between patients and controls. Isolated univariate outliers with *z*-scores greater than ±3.29 were truncated to one increment above the next largest non-outlying value.^49^ Analysis of covariance (ANCOVA) models assessed group differences in baseline impulsivity outcomes, controlling for demographic variables. Where standardized residuals were not normally distributed (Shapiro-Wilk tests) or equality of variance was violated (Levene's test), logarithm (base 10) transformations were applied to dependent variables (UPPS-P “premeditation” and “perseverance,” DDT “Mazur's k,” SWM “between-errors” and SSRT) before analysis. Where transformations were unsuccessful (BIS “motor,” IST “fixed-win” and “decreasing-win,” and AGN “positive words” and “negative words”), results were reported using robust (Huber-White) estimation. Effect sizes for ANCOVAs were calculated using partial-eta squared (*η*^2^), where .01, .06, and .14 indicate small, medium, and large effect sizes, respectively.^50^ Paired samples *t*-tests assessed changes in patient data between baseline and follow-up. Non-normally distributed data (Shapiro–Wilk tests; DDT “Mazur's k” and SSRT) were log (base 10) transformed before analysis. A Wilcoxon signed-rank test was used for IST “decreasing-win” values as they could not be corrected. To test for sampling bias, independent samples or Mann–Whitney *U*-tests examined differences in baseline impulsivity outcomes between patients who did vs. did not complete the follow-up session. Logistic regressions were performed on discharge status (discharge against medical advice vs. planned treatment termination/completion) for each self-report and behavioural baseline impulsivity outcome separately. Post hoc Bonferroni tests were used to correct for multiple comparisons. The Wilcoxon test was conducted using R statistical software version 4.4.1. All other analyses were computed using SPSS version 29.0 (IBM, Armonk, NY).

## Results

### Participants

Demographic characteristics and patient diagnoses are displayed in Table 1. Patients were older and had fewer years of education than controls, and their group comprised more males. In patients, the prevalence rate of disorders according to diagnostic categories was as follows: 46% psychotic, 43% mood, 14% anxiety or stress-related, 11% post-traumatic stress, and 7% attention-deficit/hyperactivity disorder. Over 80% of patients reported >1 substance use disorder. Patient substance use data are shown in Table 2.

**Table 1.** Demographic Characteristics and Patient Diagnoses.

	Patients	Controls
*N*	56	50
Age	38.2 ± 11.7*	31.9 ± 10.0
Sex (male)	69.6%*	50%
Education (years)^a^	10.8 ± 2.8*	16.5 ± 2.9
**Race/Ethnicity**	***N* (%)**	***N* (%)**
White	41 (73)	20 (40)
Indigenous	10 (18)	1 (2)
Black	1 (2)	1 (2)
Asian	2 (4)	25 (50)
Latinx	1 (2)	2 (4)
**Substance use disorders**	***N* (%)**	
>1 disorder	46 (82)	
Alcohol only	5 (9)	
Methamphetamine only	1 (2)	
Subthreshold	4 (7)	
**Mental disorders**	***N* (%)**	
Psychotic disorders	26 (46)	
Schizophrenia/schizoaffective/unspecified	14/3/9	
Mood disorders	24 (43)	
Bipolar/depressive/unspecified	9/8/7	
Anxiety disorders	8 (14)	
Social/generalized/unspecified	2/1/5	
PTSD	6 (11)	
ADHD	4 (7)	

Note: Data presented as means ± SD, except where otherwise specified. *P < .05.

^a^ Controls: *n* = 46, Patients: *n* = 52, due to missing data.

ADHD = attention-deficit/hyperactivity disorder; PTSD = posttraumatic stress disorder.

**Table 2.** Substance Use in Patients.

	Patients		Patients *(cont’d)*
**Polydrug**		**Methamphetamine**	
Lifetime any use (*n*)^a^	46	Lifetime any use (*n*)^a^	31
Age onset^b^	16.0 ± 5.5	Age onset^b^	23.3 ± 10.3
Years used^c^	15.3 ± 11.8	Years used^c^	7.3 ± 7.0
Past 30-day user (*n*)^d^	32	Past 30-day users (*n*)^d^	11
Days used^e^	14.4 ± 11.5	Days used^e^	12.3 ± 9.5
**Alcohol**		**Heroin**	
Lifetime any use (*n*)^a^	50	Lifetime any use (*n*)^a^	34
Age onset^b^	12.1 ± 3.5	Age onset^b^	25.6 ± 9.9
Years used^c^	18.7 ± 12.7	Years used^c^	7.4 ± 10.0
Past 30-day user (*n*)^d^	29	Past 30-day users (*n*)^d^	13
Days used^e^	9.6 ± 9.9	Days used^e^	7.7 ± 9.0
**Cigarettes**		**Other opioids**	
Lifetime any use (*n*)^a^	40	Lifetime any use (*n*)^a^	26
Age onset^b^	14.2 ± 6.1	Age onset^b^	25.5 ± 9.6
Years used^c^	22.3 ± 12.2	Years used^c^	4.8 ± 5.6
Past 30-day user (*n*)^d,f^	25	Past 30-day user (*n*)^d^	17
Less than 10/day	60%	Days used^e^	9.6 ± 7.6
11–20/day	24%	**Sedatives/tranquilizers**	
21+/day	16%	Lifetime any use (*n*)^a^	23
**Cannabis**		Age onset^b^	19.4 ± 5.5
Lifetime any use (*n*)^a^	49	Years used^c^	8.3 ± 8.7
Age onset^b^	13.4 ± 3.0	Past 30-day user (*n*)^d^	14
Years used^c^	18.0 ± 13.5	Days used^e^	20.2 ± 10.2
Past 30-day user (*n*)^d^	28		
Days used^e^	13.57 ± 11.0		
**Crack/Cocaine**			
Lifetime any use (*n*)^a^	49		
Age onset^b^	23.4 ± 7.5		
Years used^c^	8.9 ± 8.4		
Past 30-day user (*n*)^d^	25		
Days used^e^	8.8 ± 9.4		

*Note*: Data presented are means ± SD, except otherwise specified. Number of days used in the past 30 days underestimates the average use per month due to overlap with days in treatment.

^a^ Data from patients who reported lifetime ≥1x use of the substance.

^b^ Polydrug: *n* = 34; alcohol: *n* = 43; cigarettes: *n* = 39; cannabis: *n* = 40; crack/cocaine: *n* = 39; methamphetamine: *n* = 31; heroin: *n* = 27; other opioids: *n* = 19; sedatives/tranquilizers: *n* = 15, due to missing data.

^c^ Polydrug: *n* = 53; alcohol: *n* = 55; cigarettes: *n* = 40; cannabis: *n* = 55; crack/cocaine: *n* = 55; methamphetamine: *n* = 43; heroin: *n* = 55; other opioids: *n* = 55; sedatives/tranquilizers: *n* = 55, due to missing data.

^d^ Data from patients who reported past 30 days ≥1x use of the substance.

^e^ Polydrug: *n* = 54; alcohol: *n* = 55; cigarettes: *n* = 31; cannabis: *n* = 55; crack/cocaine: *n* = 55; methamphetamine: *n* = 35; heroin: *n* = 55; other opioids: *n* = 55; sedatives/tranquilizers: *n* = 55, due to missing data.

^f^ Data based on responses on Fagerstrom Test for Nicotine Dependence^69^ (*n* = 25).

### Self-Report and Behavioral Impulsivity Between Patients and Controls

**Figure 1.**
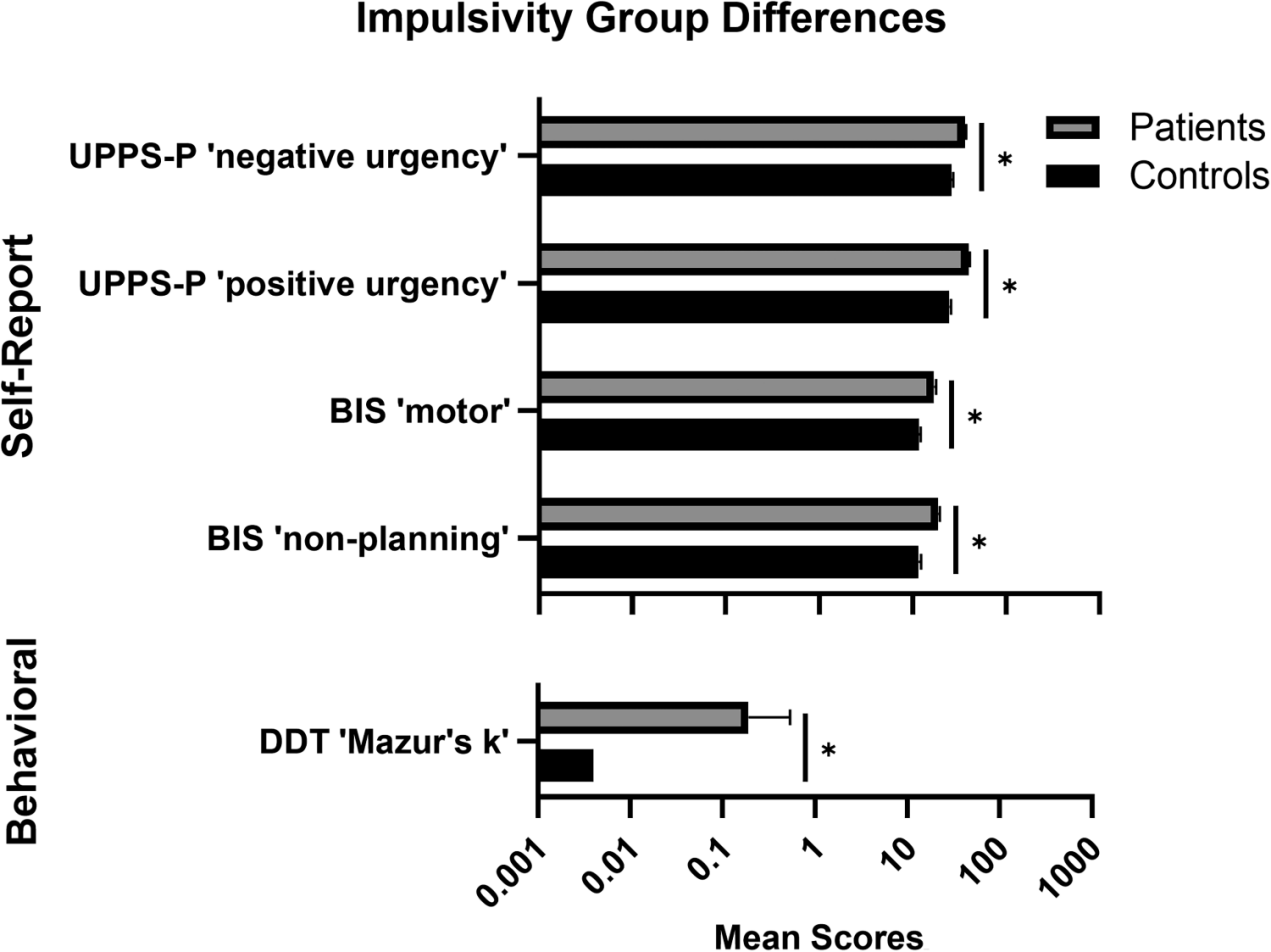
Baseline impulsivity measures in CD patients undergoing treatment vs. healthy controls. Top: Self-report impulsivity outcomes (patients: *n* = 52; controls: *n* = 46). UPPS-P = UPPS-P Impulsive Behavior Scale, BIS = Barratt Impulsiveness Scale. Bottom: Behavioral impulsivity outcomes. DDT = Delay Discounting Task (patients: *n* = 48; controls: *n* = 45). Data are means and standard errors. *Significant after Bonferroni correction (*P *< .003).

Group differences in impulsivity scores are summarized in Table 3. All ANCOVAs covaried for age, sex, and years of education. Patients had higher self-reported impulsivity than controls on most (“negative urgency,” “positive urgency,” “premeditation,” and “sensation seeking”) UPPS-P subscales, all BIS subscales, and DERS “impulse” (*P*'s < .05). Patients had higher impulsive decision-making on DDT “Mazur's k” and more working memory errors on SWM than controls (*P*'s < .05). No other behavioural group differences were observed. After Bonferroni correction to adjust for multiple comparisons (α = .003), UPPS-P “negative urgency” and “positive urgency,” BIS “motor” and “non-planning,” and DDT “Mazur's k” remained significant (Figure 1). Large effect sizes (*η*^2^≥ .14) were observed for UPPS-P “negative urgency” and “positive urgency,” BIS “motor,” and DDT “Mazur's k.”

**Table 3.** Group Differences in Impulsivity Measures – ANCOVA Results.

	Controls	Patients			
	Mean ± SEM	Mean ± SEM	Statistic	*P*	*η* ^2^
**Self-report measures**					
UPPS-P “negative urgency”	26.00 ± 1.18	36.10 ± 1.06	*F*_1,93 _= 16.09	**<.001**	.147
UPPS-P “premeditation”^a^	19.39 ± .81	23.95 ± 1.10	*F*_1,93 _= 4.10	.046	.042
UPPS-P “perseverance”^a^	18.85 ± .75	22.89 ± .81	*F*_1,93 _= 3.62	.060	.037
UPPS-P “sensation seeking”	30.54 ± 1.13	35.38 ± 1.05	*F*_1,93 _= 6.76	.011	.068
UPPS-P “positive urgency”	24.63 ± 1.24	39.52 ± 1.42	*F*_1,93 _= 24.29	**<.001**	.207
BIS “attention”	8.77 ± .53	11.62 ± .65	*F*_1,93 _= 4.20	.043	.043
BIS “motor”^b^	11.65 ± .55	16.96 ± .77	*t*_97 _*= *-4.13	**<.001**	.155
BIS “non-planning”	11.61 ± .66	18.72 ± .79	*F*_1, 93 _= 11.10	**.001**	.106
DERS “impulse”	12.39 ± .80	16.63 ± .90	*F*_1,93 _= 6.03	.016	.061
**Behavioral measures**					
DDT “Mazur's k”^a^	.004 ± .001	.19 ± .05	*F*_1,88 _= 14.52	**<.001**	.142
IST “fixed-win”^b^	.86 ± .01	.78 ± .01	*t*_89 _= 1.65	.103	.031
IST “decreasing-win”^b^	.82 ± .01	.75 ± .02	*t*_89 _= 1.81	.074	.037
AGN “positive words”^b^	504.11 ± 9.76	526.50 ± 14.81	*t*_90 _= -1.36	.178	.021
AGN “negative words”^b^	512.26 ± 9.84	519.50 ± 15.22	*t*_90 _= .45	.652	.002
SWM “between-errors”^a^	17.35 ± 2.55	41.83 ± 3.52	*F*_1,83 _= 6.10	.016	.068
CGT “risk-taking”	.58 ± .02	.63 ± .02	*F*_1,87 _= .24	.625	.003
SSRT^a^	177.57 ± 7.62	215.22 ± 8.66	*F*_1,87 _= 3.94	.050	.043

*Note*: Analysis of Covariance (ANCOVA) results are adjusted for group differences (age, sex, years education). Bolded *P*-values indicate significant results after Bonferroni correction (α = .003). Effect size (*η*^2^*)*: .01 = small, .06 = medium, .14 = large.

^a^ Log (base 10) transformation performed on data before analysis.

^b^ Huber-White standard errors used in analysis.

UPPS-P = UPPS-P Impulsive Behavior Scale, BIS = Barratt Impulsiveness Scale, DERS = Difficulties in Emotion Regulation Scale, DDT = Delay Discounting Task, IST = Information Sampling Task, AGN = Affective Go/No-Go, SWM = Spatial Working Memory, CGT = Cambridge Gambling Task, SSRT = Stop Signal Reaction Time.

### Changes Across Treatment and Treatment Outcome in Patients

Changes in measures from baseline to 6-month follow-up are summarized in Table 4. Patients were faster to inhibit responses on the SST (*P *= .024) at follow-up, but no other behavioural changes were observed. Comparisons in baseline outcomes between follow-up completers vs. non-completers revealed slower SSRT in completers (Mann-Whitney Uc = 435.0; *P *= .036), with no other differences. After Bonferroni correction (α = .006), treatment-related changes and baseline differences in SSRT were no longer significant. Binary logistic regressions revealed no significant relation of any impulsivity or working memory measure to discharge status.

**Table 4.** Treatment-Related Changes in Behavioral Impulsivity Measures in Patients.

	Baseline	Follow-Up		
	Mean ± SEM	Mean ± SEM	Statistic	*P*
DDT “Mazur's k”^a^	.25 ± .08	.61 ± .38	*t*_25 _= 1.60	.123
IST “fixed-win”	.77 ± .01	.79 ± .01	*t*_26 _= -1.59	.124
IST “decreasing-win”^b^	.75 ± .02	.76 ± .02	*z *= -.04	.981
AGN “positive words”	544.38 ± 19.33	531.32 ± 15.02	*t*_23 _= .89	.382
AGN “negative words”	538.63 ± 19.23	527.56 ± 15.02	*t*_23 _= .70	.489
SWM “between-errors”	42.27 ± 4.42	43.27 ± 4.30	*t*_25 _= -.30	.770
CGT “risk-taking”	.66 ± .03	.67 ± .03	*t*_23 _= -.36	.721
SSRT^a^	238.90 ± 13.35	216.16 ± 14.50	*t*_24 _= 2.41	.024

*Note:* Results are obtained from paired-samples *t*-tests, unless otherwise indicated. No results were significant after Bonferroni correction (α = .006).

^a^ Log (base 10) transformation performed on data before analysis.

^b^ Wilcoxon signed-rank test; *n* = 27.

Baseline = within first 2 weeks of treatment admission, Follow-up = 6 months into treatment; DDT = Delay Discounting Task, IST = Information Sampling Task, AGN = Affective Go/No-Go, SWM = Spatial Working Memory, CGT = Cambridge Gambling Task, SSRT = Stop Signal Reaction Time.

## Discussion

This study examined (1) baseline impulsivity and working memory differences between patients and controls, (2) treatment-related changes in impulsivity and working memory in CD patients from the first two weeks of admission (baseline) to 6 months of continuous care (follow-up), and (3) whether domains of functioning could predict treatment adherence. On self-reported impulsivity measures, patients scored higher on UPPS-P “negative urgency” and “positive urgency” and on BIS “motor” and “non-planning.” For behavioural outcomes, patients had higher delay discounting than controls. In patients, there were no changes over treatment on measures of behavioural impulsivity or working memory and no impulsivity measure predicted treatment adherence.

Congruent with prior research on substance use,^7,8^ mental disorder,^9^ and CD^14^ populations, patients self-reported higher impulsivity levels. Large effect sizes were observed for negative and positive urgency, along with motor impulsivity, suggesting they may represent key trait impairments in CD. There were no group differences in emotion regulation difficulties with impulse control, despite its common implication in SUDs.^51^ Exploratory evidence suggests these difficulties in CD may only arise with multiple comorbid psychiatric disorders.^52^ While testing for this was beyond the scope of our study, future research should explore this further. Greater delay discounting was observed in patients with a large effect size, providing further support for impulsive choice impairment in SUDs^8^ and CD^15,53^ groups. On the other hand, risky decision-making, while reliably observed in substance users relative to controls,^8^ was not observed in this study. This is consistent with previous work in CD,^54,55^ and our previous work suggests that computational modelling may be necessary to uncover more latent patterns of risky choice in this population.^55^ Our data is the first to show an absence of group differences between CD and controls in reflection impulsivity, which has previously been reported to be higher in substance users relative to controls.^28^ Moreover, patients did not differ from controls on working memory performance. While working memory deficits have been well-established in SUDs,^56^ conflicting results found in CD^16^ highlight the need for further investigation. Notably, there was an absence of group differences in all measures of impulsive action. Consistent with this, a recent meta-analysis^57^ reported weak evidence for response inhibition impairment in SUDs. This pattern, however, is mixed thus far in CD.^54,58,59^ More work is warranted to assess response inhibition in this population. Nonetheless, these findings indicate impulsivity as a trait marker for CD and highlight impulsive choice as a potential target area for CD treatment.

In patients, there was an absence of treatment-related changes in working memory and behavioural impulsivity. Contrary to this finding, evidence suggests that extended periods of abstinence and treatment can lead to the recovery of some neuropsychological functions in substance use^60^ and mental health^61,62^ groups. Similarly, SUD treatment-related improvements in response inhibition have been reported.^63^ While the absence of changes in delay discounting over treatment is consistent with substance use populations,^63–65^ evidence for risky decision-making is mixed. It has shown to improve in a study on polysubstance-dependent,^65^ but not CD inpatients.^55^ Discrepancies with working memory and behavioural impulsivity measures may reflect slower or decreased recovery of these cognitive functions with comorbid diagnoses, as supported by research showing less recovery of cognitive functioning in CD than those with a SUD alone after treatment.^66^ Nonetheless, findings of minimal or no enhancement of behavioural impulsivity or working memory as a function of treatment may suggest conventional treatments are neglecting to target a potentially transdiagnostic process that is likely contributing to the often-protracted substance use and mental health complications.^17^ More research is needed to assess treatment-related changes in neurocognitive functioning in CD over more extended periods of abstinence.

Measures of impulsivity and working memory were not found to predict treatment outcomes in CD patients. By contrast, some studies have found working memory deficits to be associated with substance use^30,31,56^ and mental disorder^67^ outcomes. Self-reported impulsivity is well established for predicting outcomes across substance use disorders,^7,8^ while the evidence for behavioural impulsivity appears mixed. A recent review suggests that impulsive choice, but not response inhibition, reliably predicts treatment- and substance use-related outcomes in people who use substances.^27^ Discrepancies in our findings may be in part attributable to methodological variability, including differences in study outcomes. While the present study assessed treatment adherence, previous investigations have focused predominantly on abstinence or frequency of substance use^7,27^ and symptom reduction in psychiatric disorders.^9^ Further investigations are needed to assess impulsivity as it relates to various treatment outcomes in CD.

## Limitations

This study is not without limitations. Firstly, due to the heterogeneity of clinical characteristics in our population, with variation in the combination of disorders, it remains unclear whether the results are influenced by specific diagnoses. However, our study objective was to assess impulsivity in CD as a single clinical group, where the differentiation of outcomes by diagnoses was beyond its scope. Moreover, evidence increasingly identifies impulsivity as a transdiagnostic marker for a wide range of psychiatric disorders and SUDs.^8^ Nonetheless, this area warrants further exploration. Second, because our focus was on investigating changes in impulsivity under treatment “as usual,” we did not assess active psychiatric medications and changes to the severity of the patient's psychiatric symptoms. It is unclear whether overall mental health status may have contributed to the results, such as those related to treatment outcomes (e.g., adverse medication effects leading to premature dropout). Third, patient and control groups were not demographically matched. While we controlled for demographic differences, their potential influence on impulsivity outcomes is not fully accounted for. Fourth, the attrition rate at the follow-up was high (52%), therefore, results may not generalize to our entire study population. As baseline impulsivity measures did not differ between follow-up completers vs. dropouts, our results suggest that sample bias is unlikely. Nonetheless, results should be regarded as preliminary and warrant confirmation by future studies. Fifth, we did not assess participant income status in examining differences in delay discounting. Given that income has shown to be associated with delay discounting performance,^68^ it should be included in future studies with CD. Sixth, our sample did not have enough females to conduct subgroup comparisons; therefore, our findings do not provide insights into sex differences. These differences should be explored in future studies. Finally, treatment facilities may vary in a multitude of ways, from the availability of resources to their model of care. Our results were obtained in a sample of inpatients from a single facility, which may limit the generalizability of the findings to the broader population of CD inpatients.

## Conclusions

Limitations notwithstanding, this is the first study to investigate changes in impulsivity and working memory in CD as a function of treatment. Our data suggests that impairments in some areas of trait and choice impulsivity may serve as key targets for advancing interventions for the CD clinical population. Although we observed no temporal change across domains of impulsivity or working memory, and no association with treatment adherence, future investigations of the transdiagnostic features in these areas and their influence on outcomes are warranted.
